# Promote Health or Prevent Disease? The Effects of Health-Related Advertising on Eating Behavior Intention

**DOI:** 10.3390/ijerph120403517

**Published:** 2015-03-27

**Authors:** Chia-Yen Lin

**Affiliations:** Department of Public Administration Management, National University of Tainan, 33, Sec. 2, Shu-Lin St., Tainan 700, Taiwan; E-Mail: chiayen@mail.nutn.edu.tw; Tel.: +886-62-133-111 (ext. 905); Fax: +886-62-144-409

**Keywords:** temporal distance, regulatory focus, construal level, attitude toward advertising, behavior intention

## Abstract

The health medical costs of colorectal cancer are increasingly higher in Taiwan. The National Health Insurance Administration (NHI) and The Health Promotion Administration of the Ministry of Health and Welfare (MOHW) in Taiwan encourage individuals to adopt an earnest approach to healthy behavior through advocacy advertising. However, the number of colorectal cancer patients continues to increase annually. Our study explored the effects of health-related advertisements (ads) on healthy behavior intentions as influenced by regulatory focus theory (RFT) and construal level theory (CLT). We conducted an experiment with different public health advocacy ads. A 2 (regulatory focus: promotion *vs.* prevention) × 2 (temporal distance: one month *vs.* one year) × 2 (graphics-text ratio: more pictures and less text *vs.* fewer pictures and more text) three-factor experiment was adopted. The multiple analysis of variance (MANOVA) results revealed that ads with higher construal levels (*i.e.*, more text) had greater effects with a promotion-oriented regulatory focus. However, no significant differences were found in either attitude toward the ads or behavior intention when the regulatory focus was prevention. In addition, according to the young testers and those who were psychologically distant from colorectal cancer, different temporal distances and different construal levels had no statistically significantly effects on attitudes toward advertising or on behavior intentions. The results revealed that viewers found the information easier to understand when the ads triggered the regulatory focuses of the viewers and applied an appropriate graphics-text ratio, which resulted in favorable health-related advertising effectiveness. Thus, we provide two suggestions regarding the use of health-related advertising for MOHW in the future.

## 1. Introduction

Researchers believe that 30% of the causes of cancer are dietary, and the instances of cancer in Western societies are thought to be related to Westerners’ customary intake of animal products, fats, and carbohydrates [1]. According to the National Health Insurance Administration (NHI), colorectal cancer is a catastrophic illness in Taiwan, and the government has been paying for the treatment of this illness increasingly often in recent years. One way to slow the increase in health-care costs is to change health-related behavior. Research results have found a close relationship between diet and colorectal cancer prevention. To urge the public to improve its health by eating a healthy diet and to advocate the idea that colorectal cancer can be effectively prevented by reducing potential dietary risk factors, a number of colorectal cancer prevention and cure associations currently use advertisements (ads) as a means of communication (The Colorectal Cancer Care Network, The Health Promotion Administration of the Ministry of Health and Welfare (MOHW), The Taiwan Colorectal Cancer Alliance, *etc.*). The MOHW encourages individuals to follow a healthy diet to prevent colorectal cancer. However, the number of patients who are diagnosed with colorectal cancer continues to increase annually in Taiwan. According to the report by the MOHW, colorectal cancer is the most common malignancy in Taiwan [2]. The incidence of colorectal cancer in Taiwan increased rapidly from 13.84% in 2008 to 15.20% in 2011, and the number of patients reached 14,807 in 2011, compared with 11,351 in 2008 [3]. The government’s existing colorectal cancer-related advertising materials have not attained the goal of promoting healthier eating behavior. In a review of the colorectal cancer-related advertisements produced by the government [4], we found that the information focused on “colorectal cancer screening (or monitoring)” or “colorectal cancer warning signs” and that the audience targeted by these ads was the elderly. We infer that this focus is why the number of patients is increasing annually and the average age of patients is younger than it was previously. It follows that the increasing incidence of colorectal cancer in Taiwan may be closely related to the fact that government’s advertising content has not had the expected effect. Therefore, understanding the message content in effective ads is of the utmost importance. These associations failed to consider their viewers’ mental states and make necessary adjustments to the content of the ads, leading to ineffective results. Noting different people’s psychological mind-sets to increase advertising’s persuasiveness is an area that remains unexplored. 

In the field of psychology, regulatory focus theory (RFT) and construal level theory (CLT) have been proposed in recent years. To date, numerous studies have applied RFT [5,6,7] or CLT [8,9] to measure the communication effectiveness of health messages. However, there are no empirical studies that combine RFT and CLT and demonstrate their ability to improve food ads and ads that promote good health. A number of studies suggest that a regulatory focus can improve the communication effect of health-related messages [5,10,11]. Other studies have demonstrated that different construal levels have different effects on perceptions regarding health risk judgment or medical decision making [9,12]. Although researchers have revealed that different temporal distances (*i.e.*, near *vs.* distant) with respect to purchase decisions affected preferences regarding regulatory focus product information, empirical evidence for the persuasive effect of the fit between RFT and CLT on messages regarding good health remains lacking [13].

The aim of this current study is to identify strategies to improve the persuasiveness of healthy promotion ads to change people’s eating behaviors. An experiment was conducted to improve the strategies used to develop health-related ads, that is, to create highly persuasive messages using CLT and RFT to enable viewers to consider the messages and change their behavior.

### Literature and Hypotheses

#### Regulatory Focus Theory (RFT) and Construal Level Theory (CLT)

RFT states that the focus is promotion when people focus on their ideals and the focus is prevention when people regard their goals as duties or responsibilities that should be fulfilled [9]. Scholars have discovered that participants can be manipulated into being promotion-focused by asking them to recall hopes as well as duties, confirming that situational regulatory focus can be manipulated [13]. Although people want to achieve favorable results by making correct decisions, their regulatory orientations and the strategies they employ to achieve their goals differ. People with a promotion focus prefer to use eager strategies, that is, strategies that facilitate positive outcomes. Those individuals with a prevention focus prefer vigilant strategies, which facilitate non-negative outcomes [14]. When people’s strategies match their regulatory orientations, they have a regulatory fit, and regulatory fit adds value to people’s actions, increasing their motivation to achieve their goals [15,16], positive feelings about their ideal choices, and negative feelings about non-ideal choices [17]. 

CLT suggests that people adopt high-level construals that are more abstract, general, superordinate, and decontextualized for events with greater psychological distance [18]. Conversely, for events with closer psychological distance, people adopt low-level construals that are more concrete, specific, and subordinate. CLT proposes that four psychological distances influence how people process information. Of the four psychological distances, temporal distance was the first to be introduced. One study determined that people focus more on the “big picture” rather than small details when the temporal distance is far into the future [19]. Another study compared the effects of a longer (*i.e.*, telling participants that a great number of people contract mononucleosis every year) *vs.* a shorter (*i.e.*, telling participants that a great number of people contract mononucleosis every day) time frame on participants’ feelings that they were likely to contract a disease; when the time frame is days, people can more effectively visualize contracting the disease [20]. Therefore, with messages that incorporate a short time frame, viewers’ perceptions of their risks increase, and they are more likely to engage in disease prevention. 

#### Graphics-Text Ratio

All messages can be presented in the form of graphics or text. Graphics and text feature different qualities: graphics are analogical and enable people to understand ideas quickly by looking at them, whereas text is symbolic and can present abstract information on topics of concern as well as illustrate the nature of things. These qualities show how graphics are similar to low-level construals, whereas text is similar to high-level construals [20]. Using CLT, we hypothesized that ads should be presented in a concrete format (*i.e.*, consist of many graphics and little text) for near temporal distances to enable participants to feel a fit between the presentation method and the psychological distance. Conversely, for farther temporal distances, ads should be presented in an abstract format (*i.e.*, a great deal of text and few graphics) to enable participants to feel that same fit. We proposed the following hypotheses:

Hypothesis 1 (H1): When the temporal distance between the present and the time portrayed in the advertisement (ad) is short, viewer attitudes toward the ad are more positive when the ad is concrete (*i.e.*, many graphics and little text) than when the ad is abstract (*i.e.*, a great deal of text and few graphics). Conversely, when the temporal distance between the present and the time portrayed in the ad is longer, viewer attitudes toward the ad are more positive when the ad is abstract.

Hypothesis 2 (H2): When the temporal distance between the present and the time portrayed in the ad is near, viewer behavioral intentions increase when the ad is concrete as opposed to abstract. Conversely, when the temporal distance between the present and the time portrayed in the ad is distant, viewer behavioral intentions increase when the ad is abstract as opposed to concrete.

RFT asserts that the goals of people with a prevention focus primarily consist of safety and responsibility. These individuals are more concerned with the feasibility of achieving a goal, and they focus on how to achieve it [21]. This relationship is similar to low-level construal, in which people process specific and concrete information and focus on the feasibility and methods of achieving their goals. In contrast, the goals of people with a promotion focus primarily comprise growth and advancement. They are more concerned with the ideal method for achieving a goal and how to achieve it, similar to high-level construal, in which people process abstract information and focus on how to achieve their goals [9]. Research results revealed that when a purchase decision is about to be made, consumers prefer prevention- (*vs.* promotion-) framed products. However, when a purchase is temporally distant, promotion- (*vs.* prevention-) framed products become more appealing. Therefore, people with a promotion focus are more likely to adopt high-level construal when processing information [22,23]. In the present study, we learned that a high-level construal approach should be adopted for ads aimed at people with a promotion focus, with more text and fewer graphics. In contrast, low-level construal should be adopted to design ads for those people with a prevention focus, that is, many graphics and little text. These tailored approaches will enable participants to perceive the fit between the presentation method and the regulatory focus. Therefore, we proposed the following hypotheses:

Hypothesis 3 (H3): When the regulatory focus in an ad is promotion, viewer attitudes toward the ad are more positive when the ad is abstract rather than concrete. Conversely, when the ad’s regulatory focus is prevention, viewer attitudes toward the ad are more positive when the ad is concrete rather than abstract.

Hypothesis 4 (H4): When the regulatory focus in an ad is promotion, viewer behavioral intentions increase when the ad is concrete rather than abstract. Conversely, when the ad’s regulatory focus is prevention, viewer behavioral intentions increase when the ad is abstract.

#### Attitude toward Advertising and Eating Behavior Intention

Attitude is a notable concept in psychology and is applied in sociology and consumer behavior. Researchers have argued that attitude entails a person’s evaluation of, emotion toward, or actions executed regarding an idea or thing. In this study, attitudes toward the ads were measured using the dimensions of perception and emotion. Studies have revealed a positive relationship between attitudes toward ads and purchase intentions [24,25]. Therefore, inspiring positive attitudes toward ads increases consumers’ behavioral intentions. 

Behavioral intention is the likelihood that a person will perform a specified action. A person who exhibits a strong intention to engage in a certain behavior is more likely to engage in that behavior. In health-related fields, a number of studies have been conducted that focused on changing people’s attitudes to improve their behaviors. Efforts have included ads that promote smoking cessation in adolescents [26,27], eating a healthy diet [28,29], and developing an exercise habit [30]. These studies have revealed that eating behavioral intentions and actual eating behavior are highly correlated.

## 2. Method

### 2.1. Experiment Design 

**Figure 1:**
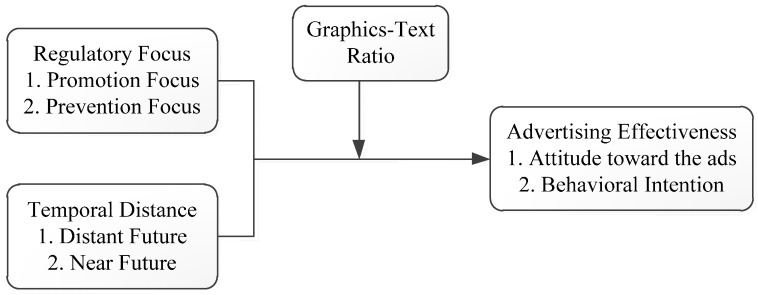
Research framework.

In this study, the independent variables were regulatory focus (*i.e.*, prevention *vs.* promotion), temporal distance (*i.e.*, one month *vs.* one year) and graphics-to-text ratios (*i.e.*, many graphics but little text *vs.* a great deal of text but few graphics; hereafter abbreviated as the graphics-text ratio). The dependent variables were attitude toward the ad and behavioral intention. We investigated the advertising effectiveness of the different regulatory focuses and temporal distances. The study framework is shown in Figure 1. 

**Figure 2:**
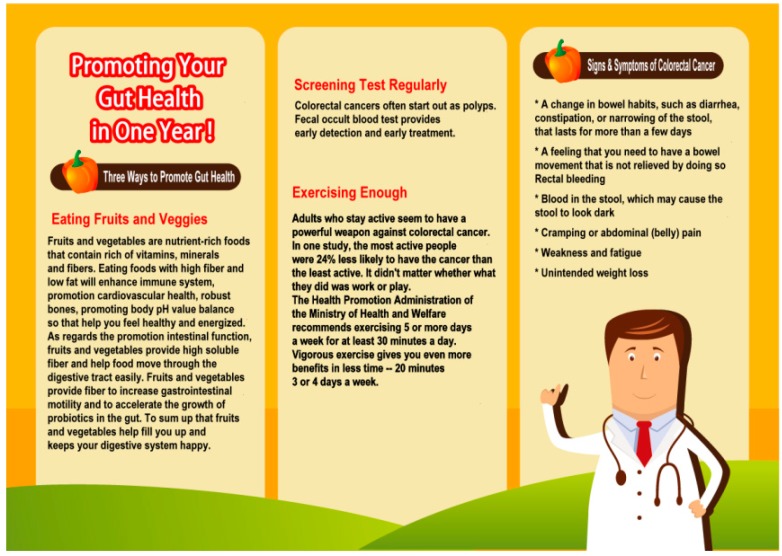
Promotion focus, one year and great deal of text and few graphics.

**Figure 3:**
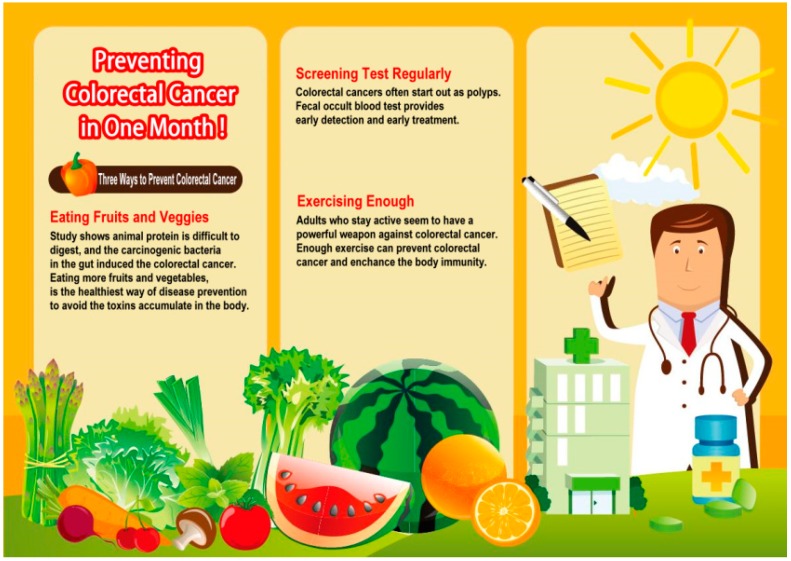
Prevention focus, one month and many graphics and little text.

A three-factor experiment was adopted, using regulatory focus (*i.e.*, promotion *vs.* prevention), temporal distance (*i.e.*, one month *vs.* one year), and graphics-text ratios (*i.e.*, many graphics and little text *vs.* a great deal of text and few graphics) for a total of eight possible scenarios. Figure 2 and Figure 3 are sample ads of the eight possible scenarios. Figure 2 is a scenario with a promotion focus, one year and great deal of text and few graphics. Figure 3 is a scenario with a prevention focus, one month and many graphics and little text. 

### 2.2. Manipulation 

To manipulate the participants’ regulatory focuses, the study incorporated a vegetable ad that had been introduced by the Ministry of Health and Welfare, in addition to the Ministry’s policies [31]. The message “promote health” was used to trigger the participants’ promotion focus, and the message “prevent disease” was used to trigger their prevention focus. We adopted the temporal distances of one month (near future) and one year (distant future) to examine the effects of varying temporal distances on promoting healthy behavior [32]. The ads were presented in the form of graphics or texts and provided as print leaflet ads. Based on a study of the traits of Chinese characters and graphics, researchers asserted that messages consisting of many graphics but little text are concrete and are characterized as low-level construals, whereas messages consisting of a great deal of text but few graphics are abstract and are characterized as high-level construals [33]. In this study, the graphics-text ratios were set as follows: for ads consisting of a great deal of text but few graphics, the text accounted for approximately two-thirds to three-fourths of the ad, and for ads consisting of many graphics but little text, the graphics accounted for approximately two-thirds to three-fourths of the ad.

Two questions were designed to check the perceptions of temporal distance and the graphics-text ratio of the subjects: (Q1) *What is the temporal distance implied in the advertisement? One month or one year?* and (Q2) *What is the graphics-text ratio in the advertisement?* *More graphics or more text?* We eliminated the participant from the sample when he or she chose a wrong answer according to our design. In addition, four other statements were designed to determine whether the regulatory focuses were successfully designed in the ads. In the promotion focus, the four questions were as follows: (Q1) *The advertisement declared eating fruits and vegetables may strengthen the immune system.* (Q2) *The advertisement declared eating fruits and vegetables can enhance skin protection.* (Q3) *The* *advertisement declared eating fruits and vegetables can accelerate the growth of probiotic bacteria.* (Q4) *The advertisement declared eating fruits and vegetables may lower cholesterol.* In the prevention focus, the four questions were as follows: (Q1) *The advertisement declared eating fruits and vegetables may prevent cancer.* (Q2) *The advertisement declared eating fruits and vegetables can enhance skin protection.* (Q3) *The advertisement declared eating fruits and vegetables can prevent the accumulation of toxins.* (Q4) *The advertisement declared eating fruits and vegetables may lower cholesterol*. 

### 2.3. Measurement of Outcome Variables

In this study, we used two independent variables to measure the communication effect of the advertisements: attitude toward the ads and the behavior intention. We consulted the previous research and adopted 11 items to measure the attitude toward the ads (*i.e.*, *Attad1: clearly expressed/Attad2: delightful/Attad3: excellent/Attad4: convincingly/Attad5: positive/Attad6: effective/Attad7: very attractive/Attad8: helpful/Attad9: important/Attad10: strongly agree/Attad11: good advice*) by asking the question “*How do you feel about this advertisement?*” [34] Scholars have defined behavioral intention as the likelihood that a person will perform a specified action. In the health promotion area, many researchers have predicted actual behavior by measuring the individual behavior intention [26,27,28]. In this study, we adopted a method proposed by Updegraff *et al.* [35] and developed six statements to measure behavior intention: (BI1) *After reading the ad, I began to be aware that gut-related disease could occur.* (BI2) *I would accept the recommendation and behave as instructed in the guidance provided by the ad.* (BI3) *I will pay more attention to the probability of gut disease in the future.* (BI4) *This ad made me pay more attention to health.* (BI5) *I am now willing to change my eating behavior and increase my intake of vegetables and fruits.* (BI6) *I would like to buy more vegetables and fruits to eat.* The attitude toward the ads and the behavior intention were measured using Likert 7-point scales (from strongly disagree to strongly agree).

### 2.4. Procedure and Samples

In this study, we use two independent variables to measure the communication effect of the ads: attitude toward the ads and the behavior intention. Participants received a print leaflet ad, which was randomly assigned to one of the eight designed scenarios. After reading the content of ads, the participants were asked to complete the questionnaire related to the attitude toward the ads and their future behavior intention. The participants were recruited from classrooms at the National University of Tainan, and the questionnaire survey carefully followed the relevant participant privacy and human research ethics policies. 

A total of 308 questionnaires were distributed to the participants. The number of valid questionnaires after removing the 92 invalid questionnaires was 216. Most of the participants were students (61.6%), the average age was 26.78 years old, and the percentage of female participants was higher (62.3%). Regarding eating behaviors, the participants ate fruits and vegetables for all three meals only 12% of the time, ate fruits and vegetables for two meals 37.1% of the time, and ate fruits and vegetables for only one meal or not at all 50.5% of the time. Table 1 shows the sample size for each scenario.

**Table 1:** Group size for each scenario.

Scenarios	Regulatory Focus	Temporal Distance	Graphics-Text Ratio	Valid Sample Size
1	Promotion	One Month	Many graphics but little text	27
2	Promotion	One Month	A great deal of text but few graphics	25
3	Promotion	One Year	Many graphics but little text	32
4	Promotion	One Year	A great deal of text but few graphics	24
5	Prevention	One Month	Many graphics but little text	29
6	Prevention	One Month	A great deal of text but few graphics	23
7	Prevention	One Year	Many graphics but little text	26
8	Prevention	One Year	A great deal of text but few graphics	30
Total				216

## 3. Results and Discussion

First, in Section 3.1, we provide the results of the manipulation test and the variable measurements. Then, in Section 3.2, the MANOVA result is reported. Section 3.3 provides a detailed discussion of the managerial implications of this study.

### 3.1. Manipulation Test and Variable Measurement Reliability 

Four questions were designed to test whether the temporal distances, graphics-text ratios and regulatory focuses were successfully designed. Samples were deleted if the temporal distance and graphics-text ratios were incorrect. A paired-samples t-test was used to analyze whether the regulatory focus had been successfully manipulated. The results revealed significant differences between manipulating the promotion focus and manipulating the prevention focus (M*_promotion_* = 5.8194 > M*_prevention_* = 4.2176, t*_(__107__)_* = 9.141, *p* < 0.001). Regarding the prevention focus, the results showed that manipulating the prevention focus achieved significantly different results than manipulating the promotion focus (M*_promotion_* = 4.1343 < M*_prevention_* = 5.8750, t*_(107)_* = 9.161, *p* < 0.001). 

A reliability analysis of internal consistency revealed that the Cronbach’s α values for attitudes toward the ad and behavioral intentions were 0.943 and 0.892, respectively after items with lower reliability coefficient value were deleted (Table 2). Both of these values were higher than 0.7, indicating that the questionnaire items were consistency of the scale.

**Table 2:** Reliability of dependent variables.

Items	Corrected Item-Total Correlation	Cronbach’s α if Item Deleted	Cronbach’s α
Attad1	0.734	0.938	0.943
Attad2	0.702	0.939	
Attad4	0.696	0.940	
Attad5	0.779	0.936	
Attad6	0.728	0.938	
Attad8	0.780	0.936	
Attad11	0.764	0.937	
BI1	0.628	0.887	0.892
BI2	0.744	0.869	
BI3	0.729	0.871	
BI4	0.784	0.863	

Note: The reliability output was calculated after deleting items with lower reliability coefficient value.

### 3.2. MANOVA Result

The results of multifactor multivariate tests showed no statistically significant differences for the main effect (Table 3). However, regarding the interaction effect between regulatory focus and the graphics-text ratios, significant differences were observed (F*_(2,207)_* = 3.458, *p* < 0.05). The results from analyzing the between-subject effects of the interactions between regulatory focus and graphics-text ratios on viewer attitudes toward the ad (F*_(1,208)_* = 4.591, *p* < 0.05) and viewer behavioral intentions (F*_(1,208)_* = 2.972, *p* < 0.05) were significant (Table 4 and Table 5). The effects of the interactions between the other independent variables on viewer attitudes toward the ad and viewer behavioral intentions were insignificant at the 0.05 significance level. According to Pandelaere *et al.* [36], a significance level of *p* < 0.1 (marginal significance) signifies that a hypothesis has management implications. Therefore, our manipulation of the graphics-text ratios and temporal distances to influence viewers’ behavioral intentions (F*_(1,208)_* = 2.610, *p* < 0.1) had management implications.

**Table 3:** Multivariate tests.

Effect	Value	F	Hypothesis df	Error df	Sig.
Regulatory focus	0.004	0.391	2	207	0.677
Graphics-text ratio	0.011	1.158	2	207	0.316
Temporal distance	0.001	0.101	2	207	0.904
Regulatory focus × Graphics-text ratio	0.032	3.458 *	2	207	0.033 *
Regulatory focus × Temporal distance	0.005	0.525	2	207	0.592
Graphics-text ratio × Temporal distance	0.019	1.953	2	207	0.144
Regulatory focus × Graphics-text ratio × Temporal distance	0.002	0.228	2	207	0.796

* *p* < 0.05.

**Table 4:** Tests of between-subject effects for attitude toward advertisment.

Source	Measure: Attitude toward Ad				
	Type III Sum of Squares	df	Mean Squared	F	Sig.
Regulatory focus	0.271	1	0.271	0.382	0.537
Graphics-text ratio	0.048	1	0.048	0.068	0.795
Temporal distance	0.094	1	0.094	0.133	0.716
Regulatory focus × Graphics-text ratio	4.591	1	4.591	6.487 *	0.012
Regulatory focus × Temporal distance	0.660	1	0.660	0.933	0.335
Graphics-text ratio × Temporal distance	1.809	1	1.809	2.556	0.111
Regulatory focus × Graphics-text ratio × Temporal distance	0.043	1	0.043	0.061	0.805
Error	147.211	208	0.708		
R-square	0.052				

* *p* < 0.05.

**Table 5:** Tests of between-subject effects for behavior intention.

Source	Measure: Behavior Intention				
	Type III Sum of Squares	df	Mean Squared	F	Sig.
Regulatory focus	0.571	1	0.571	0.760	0.384
Graphics-text ratio	1.454	1	1.454	1.936	0.166
Temporal distance	0.135	1	0.135	0.179	0.672
Regulatory focus × Graphics-text ratio	2.972	1	2.972	3.957 *	0.048
Regulatory focus × Temporal distance	0.517	1	0.517	0.689	0.407
Graphics-text ratio * Temporal distance	2.610	1	2.610	3.475 ^	0.064
Regulatory focus × Graphics-text ratio × Temporal distance	0.328	1	0.328	0.437	0.509
Error	156.228	208	0.751		
R-square	0.058				

^ *p* < 0.1, * *p* < 0.05.

**Figure 4:**
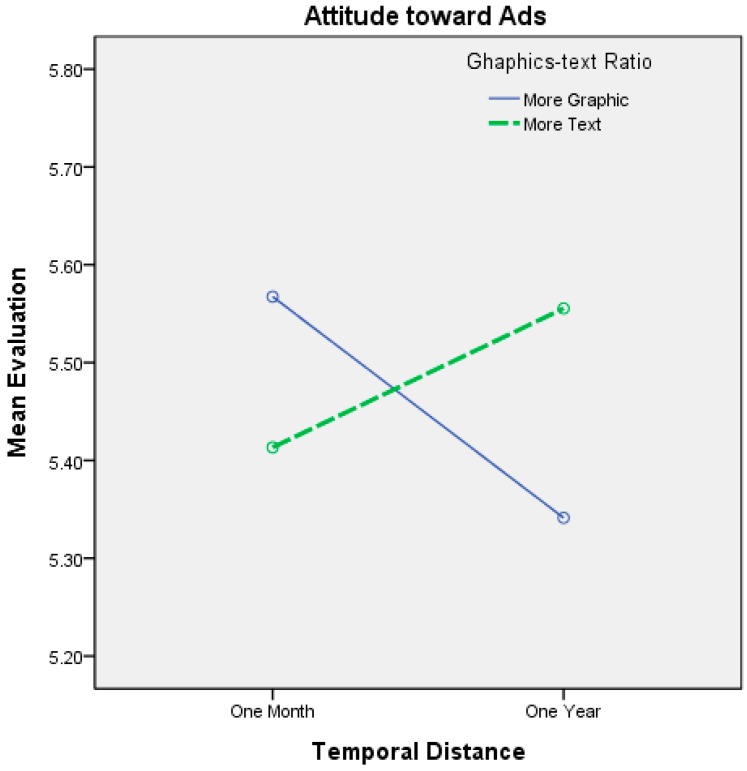
Interaction effects between temporal distance and the graphics-text ratio on attitude toward advertisement.

**Figure 5:**
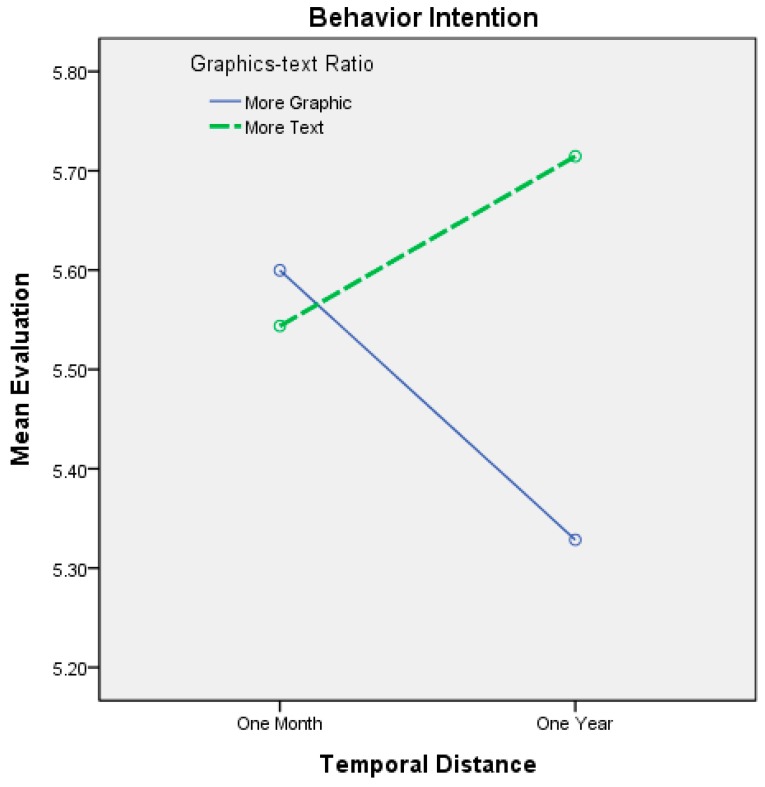
Interaction effects between temporal distance and the graphics-text ratio on behavior intention.

The interactions between temporal distance and the graphics-text ratio are shown in Table 6 and Figure 4. When the dependent variable was attitude toward the ad with one month of temporal distance, the participants’ attitudes toward the ad were more positive when the ad was concrete (*i.e.*, many graphics and little text) as opposed to when the ad was abstract. However, the difference was also not statistically significant. When the temporal distance was one year, the participants’ attitudes toward the ad were more positive when the ad was abstract (*i.e.*, a great deal of text and few graphics) as opposed to when the ad was concrete. However, this difference was not statistically significant, which directionally supports H1. Although the differences were not statistically significant for either temporal distance, the direction of the interaction effects supported our hypotheses on viewers’ attitudes toward the ad. The effects of the interactions between temporal distance and the graphics-text ratios on the participants’ behavioral intentions are shown in Table 6 and on the right side of Figure 4 and Figure 5. When the temporal distance used was one month, the participants’ behavioral intentions were higher with concrete (*i.e.*, many graphics and little text) as opposed to abstract ads; however, the difference was not statistically significant, which partially supports H2. When the temporal distance was one year, the participants’ behavioral intentions were higher when the ad was abstract (*i.e.*, a great deal of text and few graphics) as opposed to when it was concrete. This difference was significant, which supports H2. Although the difference was not statistically significant for one month, the direction of the interaction effects supported our hypotheses on viewers’ behavioral intentions.

**Table 6:** Interactions between temporal distance and the graphics-text ratio.

Independent Variables	Temporal Distance	Graphics-Text Ratio	Sample Size	Mean	S.D.	Sig. (*p*-value)
Attitude toward Ad	One Month	Many graphics but little text	56	5.5714	0.7603	0.360
		A great deal of text but few graphics	48	5.4196	0.9227	
	One Year	Many graphics but little text	58	5.3153	0.9226	0.155
		A great deal of text but few graphics	54	5.5476	0.7843	
Behavior Intention	One Month	Many graphics but little text	56	5.6027	0.8591	0.749
		A great deal of text but few graphics	48	5.5469	0.9163	
	One Year	Many graphics but little text	58	5.3017	0.8387	0.014 *
		A great deal of text but few graphics	54	5.7083	0.8765	

* *p* < 0.05.

**Figure 6:**
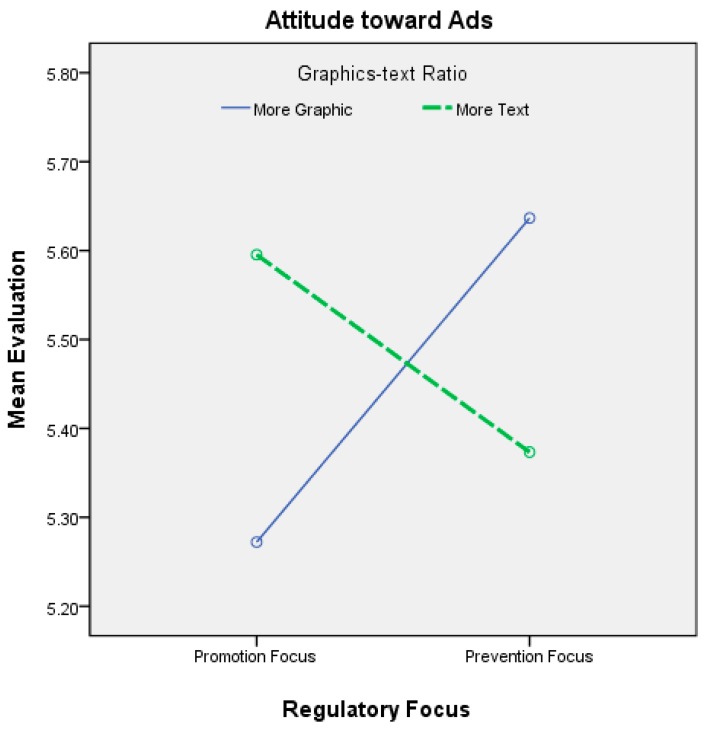
Interaction effects between regulatory focus and the graphics-text ratio on attitude toward advertisement.

**Figure 7:**
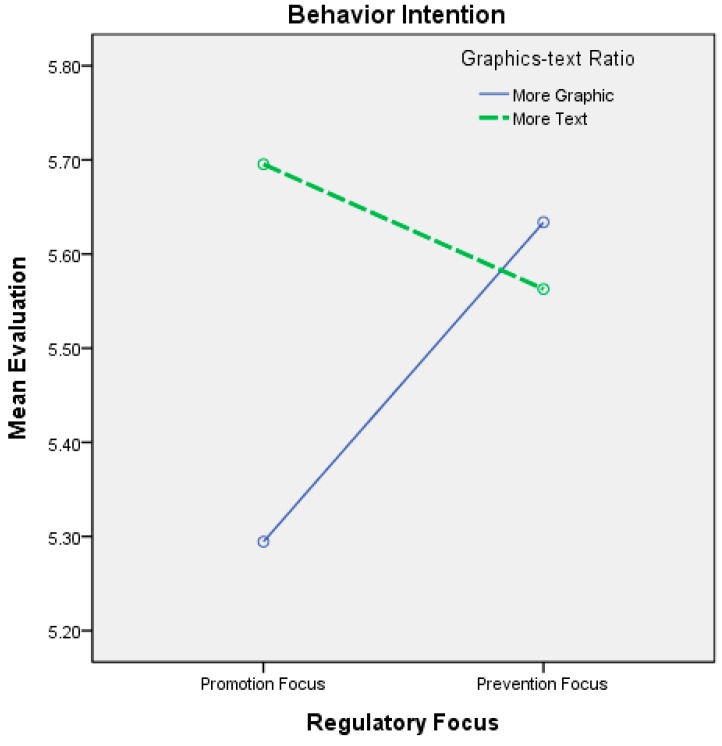
Interaction effects between regulatory focus and the graphics-text ratio on behavior intention.

The interactions between regulatory focus and the graphics-text ratio are shown in Table 7 and Figure 6 and Figure 7. When the dependent variable was attitude toward the ad and the regulatory focus was promotion, the participants’ attitudes toward the ad were more positive when the ad was abstract (*i.e.*, a great deal of text and few graphics) as opposed to when it was concrete. The difference was significant. When the regulatory focus was prevention, the participants’ attitudes toward the ad were more positive when the ad was concrete (*i.e.*, many graphics and little text) as opposed to when it was abstract; however, the difference was not statistically significant. Although the difference was not statistically significant, the direction of the interaction effect supported our hypotheses on viewers’ attitudes toward the ad.

**Table 7:** Interactions between regulatory focus and the graphics-text ratio.

Independent Variables	Regulatory Focus	Graphics-Text Ratio	Sample Size	Mean	S.D.	Sig. (*p*-value)
Attitude towards Ads	Promotion Focus	Many graphics but little text	59	5.2567	0.8940	0.039 *
		A great deal of text but few graphics	49	5.5948	0.7662	
	Prevention Focus	Many graphics but little text	55	5.6390	0.7658	0.125
		A great deal of text but few graphics	53	5.3881	0.9175	
Behavior Intention	Promotion Focus	Many graphics but little text	59	5.2754	0.8581	0.014 *
		A great deal of text but few graphics	49	5.6939	0.8843	
	Prevention Focus	Many graphics but little text	55	5.6364	0.8261	0.106
		A great deal of text but few graphics	53	5.5755	0.9088	

* *p* < 0.05.

Thus, H4 was partially supported. The effects of the interactions between regulatory focus and the graphics-text ratio on viewers’ behavioral intentions are also shown in Figure 3. When the regulatory focus was promotion, the viewers’ behavioral intentions were higher when the ad was abstract (*i.e.*, a great deal of text and few graphics) as opposed to concrete. The difference was significant, supporting the first part of H4. When the regulatory focus was prevention, the participants’ behavioral intentions were higher when the ad was concrete (*i.e.*, many graphics and little text) as opposed to when it was abstract; however, the difference was not statistically significant. Although the difference was not statistically significant, the direction of the interaction effect partially supported our hypothesis on viewers’ behavioral intentions (H4).

### 3.3. Discussion

In this study, RFT and CLT were combined and used to identify the graphics-text ratios that improve the persuasiveness of health promotion ads. The participants’ construal levels were manipulated using varying temporal distances and different regulatory focuses. The results partially confirmed the validity of H1, H2, H3, and H4.

Although the literature on disease-related studies that use CLT have found significant differences when the temporal distance differed [5,12,13], the main effect of temporal distance was not statistically significant in this current study. However, the between-subject effects of temporal distance and the graphics-text ratio on behavior intention were statistically significant. We investigated the interaction effect of temporal distance and the graphics-text ratio and found that the difference of behavior and attitude toward ads was bigger when the temporal distance was set as “in one year” than “in one month”. We surmised that this lack of significance could have occurred because the average age of participants in this study was approximately 27 years, which is too young to have sufficient knowledge regarding colorectal cancer, whereas the participants in previous studies were approximately 40 years or older [37,38]. 

The result of the between-subject effects of regulatory focus and graphics-text ratio on attitude toward the ads and behavior were statistically significant. Our results were consistent with previous research that reported that individuals with promotion focus preferred a high construal level, which indicates that they preferred abstract advertisements, and vice versa [23,37]. However, we determined that the interaction effects of regulatory focus and the graphics-text ratio were both directional and statistically significant when the manipulation was promotion focus. When the manipulation was prevention, only directional interaction effect was supported. If we recall the discussion in the preceding paragraph and previous research [9,23,25], individuals were a regulatory fit when individuals with a promotion focus preferred the abstract goal strategy, and this preference often fit with far psychological distance. The participants in our study were far distant with respect to colorectal cancer and were more fit when they obtained abstract information such that the differences between more graphics and more text are more significant in the promotion focus than the prevention focus. 

Another reason for the lack of significance might be the focus on the actual temporal distance and the psychological temporal distance. This study advocated colorectal cancer prevention. However, colorectal cancer generally occurs in individuals aged over 50 years. In this study, most of the participants were aged 27 years or younger. Thus, they were influenced by a high-level construal of colorectal cancer regardless of the temporal distances used in the advertising messages. The participants thus processed the information via high-level construal, which differed significantly from this study’s hypothesis that a low-level construal design would be appropriate for a close temporal distance. Therefore, we inferred that the effects of the interactions between regulatory focus and graphics-text ratio on participants’ attitudes toward the ad and their behavioral intentions were not statistically significant (despite showing the correct direction) because we miscalculated the temporal distances and graphics-text ratios. 

Regarding our manipulation of the regulatory focus, the results of the overall interactions showed that a regulatory focus influenced how viewers processed information. When we triggered the participants’ prevention focus, they used low-level construal to process the information. Conversely, when we triggered their promotion focus, they used high-level construal to process information. For the first group, the ads with many graphics and little text produced superior advertising effectiveness. For the second group, the ads with a great deal of text and few graphics created superior advertising effectiveness. These results supported the findings from previous studies [23,38,39]. We inferred that no statistically significant differences were observed for the prevention focus group regarding their attitudes toward the ad or their behavioral intentions because colorectal cancer generally occurs in people aged older than 50 years in Taiwan, and most of the participants in this study were aged 27 years or younger. Thus, these participants felt relatively psychologically distant from the disease, and they adopted high-level construal and the promotion focus to process information. As such, the ads with a great deal of text but few graphics were the most effective. 

## 4. Conclusions 

The mortality rate from colorectal cancer in Taiwan continues to increase. In previous colorectal cancer-related ads, the MOHW merely emphasized the importance for viewers to undergo screening for colorectal cancer, eat fruits and vegetables, and exercise, and that failure to engage in these activities could increase the likelihood of contracting colorectal cancer. The Health Promotion Administration failed to consider viewers’ mental states and make necessary adjustments to the ads’ contents. The interactions between regulatory focus and the appropriate graphics-text ratios should have produced statistical significance. In addition, our study results revealed that viewers found the information easier to understand when the ads triggered the viewers’ regulatory focuses and applied the appropriate graphics-text ratio, producing favorable advertising effectiveness. We thus offer two recommendations regarding the use of health-related advertising in the future: First, when the goal of the ad is to stimulate the public’s intention to change their lifestyles and improve their health, ads should consist of a great deal of text but few graphics with a promotion focus slogan and content. In addition, an ad’s persuasiveness can be enhanced by emphasizing the benefits that can be derived by engaging in the advertised activities. However, when the goal of the ad is to stimulate the public’s intentions to change their lifestyles and prevent colorectal cancer, ads should contain many graphics but little text with a prevention focus slogan and content. The ad’s persuasiveness can be improved by stating the negative effects that can occur from not engaging in the advertised activities. Second, based on the results of our study (*i.e.*, the effects of the interactions between regulatory focus and the graphics-text ratio on the participants’ attitudes toward the ad and their behavioral intentions were not statistically significant when the prevention focus slogan was manipulated), messages that advertise colorectal cancer prevention and target adolescents should adopt high-level construal rather than low-level construal to achieve superior advertising effectiveness. As in the scenarios used in the experiment, if the MOHW wants to encourage young adults to adopt healthier eating behaviors, it should provide more detailed textual description in ads regarding colorectal cancer and how to promote health rather than a simple slogan or a great deal of graphics without text content. 
